# Identifying responders to elamipretide in Barth syndrome: Hierarchical clustering for time series data

**DOI:** 10.1186/s13023-023-02676-8

**Published:** 2023-04-11

**Authors:** Jef Van den Eynde, Bhargava Chinni, Hilary Vernon, W. Reid Thompson, Brittany Hornby, Shelby Kutty, Cedric Manlhiot

**Affiliations:** 1 The Blalock-Taussig-Thomas Pediatric and Congenital Heart Center, Department of Pediatrics, Johns Hopkins School of Medicine, Johns Hopkins University, Baltimore, MD USA; 2 Department of Cardiovascular Sciences, KU Leuven & Congenital and Structural Cardiology, UZ Leuven, Leuven, Belgium; 3 Department of Physical Therapy, Kennedy Krieger Institute, Johns Hopkins University, Baltimore, MD USA

**Keywords:** Barth syndrome, Digital health, Hierarchical clustering

## Abstract

**Background:**

Barth syndrome (BTHS) is a rare genetic disease that is characterized by cardiomyopathy, skeletal myopathy, neutropenia, and growth abnormalities and often leads to death in childhood. Recently, elamipretide has been tested as a potential first disease-modifying drug. This study aimed to identify patients with BTHS who may respond to elamipretide, based on continuous physiological measurements acquired through wearable devices.

**Results:**

Data from a randomized, double-blind, placebo-controlled crossover trial of 12 patients with BTHS were used, including physiological time series data measured using a wearable device (heart rate, respiratory rate, activity, and posture) and functional scores. The latter included the 6-minute walk test (6MWT), Patient-Reported Outcomes Measurement Information System (PROMIS) fatigue score, SWAY Balance Mobile Application score (SWAY balance score), BTHS Symptom Assessment (BTHS-SA) Total Fatigue score, muscle strength by handheld dynamometry, 5 times sit-and-stand test (5XSST), and monolysocardiolipin to cardiolipin ratio (MLCL:CL). Groups were created through median split of the functional scores into “highest score” and “lowest score”, and “best response to elamipretide” and “worst response to elamipretide”. Agglomerative hierarchical clustering (AHC) models were implemented to assess whether physiological data could classify patients according to functional status and distinguish non-responders from responders to elamipretide. AHC models clustered patients according to their functional status with accuracies of 60–93%, with the greatest accuracies for 6MWT (93%), PROMIS (87%), and SWAY balance score (80%). Another set of AHC models clustered patients with respect to their response to treatment with elamipretide with perfect accuracy (all 100%).

**Conclusions:**

In this proof-of-concept study, we demonstrated that continuously acquired physiological measurements from wearable devices can be used to predict functional status and response to treatment among patients with BTHS.

## Background

Barth syndrome (BTHS) is a rare X-linked genetic disease which occurs in approximately 1 in 1,000,000 male live births. Typical features of BTHS are cardiomyopathy, skeletal muscle weakness, growth retardation, neutropenia, and increased urinary excretion of 3-methylglutaconic acid [1, 2]. The underlying cause of BTHS has been traced to mutations or deletions in the tafazzin (TAZ) gene [3]. TAZ encodes a mitochondrial enzyme that remodels the acyl chains of newly synthesized cardiolipin, which is a principal phospholipid in the inner mitochondrial membrane [4]. Reduced tafazzin activity negatively affects remodeling of cardiolipin, thus perturbing various cardiolipin-dependent mitochondrial functions such as organization of the respiratory chain and maintenance of cristae morphology [5].

Elamipretide is a synthetic lipophilic tetrapeptide with high cell permeability [6]. Being the first compound to target phospholipids on membranes rather than target peptides, elamipretide interacts primarily with cardiolipin in the inner mitochondrial membrane by hydrophobic or electrostatic binding [7]. Preclinical studies have demonstrated that this interaction can improve mitochondrial respiratory function and increase energy production [8–10]. In a recent randomized, double-blind, placebo-controlled crossover trial [11], the effect of elamipretide was tested in 12 subjects with BTHS. The trial results showed that a total of 48 weeks of exposure to elamipretide resulted in a significant improvement in performance on the 6-minute walk test (6MWT) and BTHS Symptom Assessment (BTHS-SA) scale, as well as increases in cardiac stroke volumes. However, not all patients responded equally well to this therapy.

The widespread availability of real-time physiological data on various health metrics such as heart rate (HR) and respiratory rate (RR) presents a significant opportunity for data mining and machine learning to answer important clinical questions [12]. As an example, a recent report demonstrated the successful implementation of a convolutional neural network to detect coronavirus disease 19 (COVID-19) on any specific day given time series data of vital signs for that day and the preceding 4 days [13]. In the present study, we examined the correlation of HR, RR, activity, and posture measured using a wearable device with functional status. We then investigated whether hierarchical clustering based on features extracted from these time series data allows to distinguish non-responders from responders to elamipretide among patients with BTHS.

## Methods

### Patient sample and data sources

This study used data collected for the phase 2/3 randomized, double-blind, placebo-controlled crossover trial followed by an open-label treatment extension that evaluated the safety, tolerability, and efficacy of subcutaneous injections of elamipretide in subjects with genetically confirmed BTHS (Clinicaltrials.gov NCT03098797). This was a sponsor-initiated, single-site trial conducted at the Johns Hopkins Hospital. The summary results of the trial have been published previously [11]. Briefly, 12 subjects with BTHS were randomized (1:1) to one of two sequence groups: 12 weeks of single daily subcutaneous doses of 40 mg elamipretide in treatment period 1 followed by 12 weeks of treatment with placebo in treatment period 2 (separated by 4-week washout period), or vice versa. In the second part of the trial, the open-label extension trial, 10 patients continued to receive elamipretide for 36 additional weeks. Two of these patients left the trial due to injection site reactions, such that complete follow-up data for all outcomes was available through 36 weeks into the second part in 8 patients.

Participants were seen at 5 clinical visits: the screening visit (Screening), the baseline visit for treatment period 1 (Base1), the 12 week visit for treatment period 1 (End1), the baseline visit for treatment period 2 (Base2), and the 12 week visit for treatment period 2 (End2). At each of these visits, the patients were provided with an AVIVO™ mobile patient management system (Medtronic Inc.). This system is a wearable device intended to continuously measure, record and periodically transmit physiological data including electrocardiography and accelerometry. Participants were instructed to wear the System for approximately 7 consecutive days after the Screening, Base1 and Base2 visits and before the End1 and End2 visits. All data from the wearable device were complete, except for two patients who had no recordings at the End2 visit.

Outcome measures included the following: 6MWT [14], Patient-Reported Outcomes Measurement Information System (PROMIS) fatigue score, SWAY Balance Mobile Application score (SWAY balance score) [15, 16], BTHS-SA, knee extensor muscle strength as measured by handheld dynamometry (HHD) [17], 5 times sit-to-stand test (5XSST), and the elevation of the monolysocardiolipin to cardiolipin ratio (MLCL:CL). Assessments were performed at the Base1, End1, and End2 visits. In the present study, groups were created through median split of these outcomes in two different ways. First, the data from Base1, End1, and End2 were merged and dichotomized, assigning each patient out of 30 observation (3 visits of 10 patients) into two groups based on their outcome value (“highest score” and “lowest score”). Second, the baseline outcome (Base1 or Base2 depending on crossover trial allocation) was subtracted from post-elamipretide outcome (End1 or End2 depending on crossover trial allocation) to isolate the effect of elamipretide; groups were then again created according to response to elamipretide (“best response to elamipretide” [later referred to as “responders”] and “worst response to elamipretide” [later referred to as “non-responders”]).

### Study design and objectives

The current study focused on examining the collected data to detect hidden structure in physiological measurements as a means of unfolding and thus describing heterogeneity in treatment response to elamipretide in patients with BTHS. For this purpose, the study first assessed whether features extracted from time series data of each of the 3 study visits (Base1, End1, and End2) could classify patients according to functional status (“highest score” and “lowest score”). Secondly, we applied hierarchical clustering to select features that can adequately distinguish “responders” from “non-responders” to elamipretide.

### Feature processing, extraction, and selection

Physiological measurements from the wearable device considered for our analysis included: minimum, maximum, and mean HR (one measurement recorded every 5 min in beats per minute); RR (one measurement recorded every 15 min in breaths per minute); activity duration defined as the time during which the patient was active within each 4-hour registration period (one measurement recorded every 4 h in seconds); activity intensity defined as the percent of target HR (220 beats per minute minus age) which was reached during activity (one measurement recorded every 4 h); and posture defined as the percent of time that the patient was standing upright (one measurement recorded every 4 h in degrees). Another measurement, workload, was calculated by multiplying activity duration and intensity measurements.

The time series of minimum, maximum, and mean HR as well as RR were split into day (7am to 10pm) and night time (10pm to 7am) series. Time series data from only the first full 3 consecutive days of each visit were considered throughout the analysis. The Python package “tsfresh” [18] was employed to implement feature engineering of the time series data and extract approximately 790 higher dimensional temporal features from each of the series. These features provide insights into the physiological variables (PVs) and their dynamics. The extracted features included autocorrelation, time series quantiles, spectral, Fourier, linear, non-linear, polynomial, wavelet and entropy, etc. that lend themselves to clustering or any other machine learning analysis. Six descriptive statistical measures including mean, variance, minimum, maximum, 25th and 75th percentiles were extracted from activity duration, activity intensity, workload, and posture measurements for each visit.

Given the large number of features generated with tsfresh package, dimensionality reduction and the selection of outcome-specific relevant features was necessary to fine-tune and achieve optimal machine learning model performance. For this purpose, the features obtained from the tsfresh package were filtered for multi-collinearity with a correlation score greater than 0.9 to obtain reliable estimates from the model. We then used SelectKBest() from scikit-learn (https://scikit-learn.org/stable/index.html), a univariate feature selection method which analyses the relationships between the features, outcome and sort features according to their p-value. Post sorting, we chose to consider only statistically significant features (p ≤ 0.05) for unsupervised cluster analysis.

### Hierarchical clustering

The selected statistically significant features were standardized and fed into agglomerative hierarchical clustering (AHC) models using Seaborn v0.11.2 [19]. A clustermap illustrates patients with similar physiological patterns mapped according to (i) functional status, in the first objective of the study, and (ii) outcome response to elamipretide, in the second objective of the study. The mapping is based on a similarity distance metric between the patients’ group identity and physiological measurements from the wearable device at each clinical visit. As a result, patients with both highly correlated outcome value response and similar PVs were clustered. We chose standardized Euclidean metric with complete linkage method for each of the developed AHC models, allowing for direct comparability. All statistical analyses were performed using Python v3.9.

## Results

### Physiological data accurately classify patients according to functional status

The first analysis was to assess whether the physiological measures from the wearable device correlated with functional status. Clustering performance was assessed with the data from 3 clinical visits (Base1, End1, and End2) of 10 patients who were screened for baseline values and received both placebo and elamipretide during the trial (2 patients with incomplete data were excluded). Outcome data from these 3 visits were merged and dichotomized with a median split, assigning the 30 observations (10 patients per visit) into two groups based on their outcome value (“highest score” and “lowest score”).

**Fig. 1:**
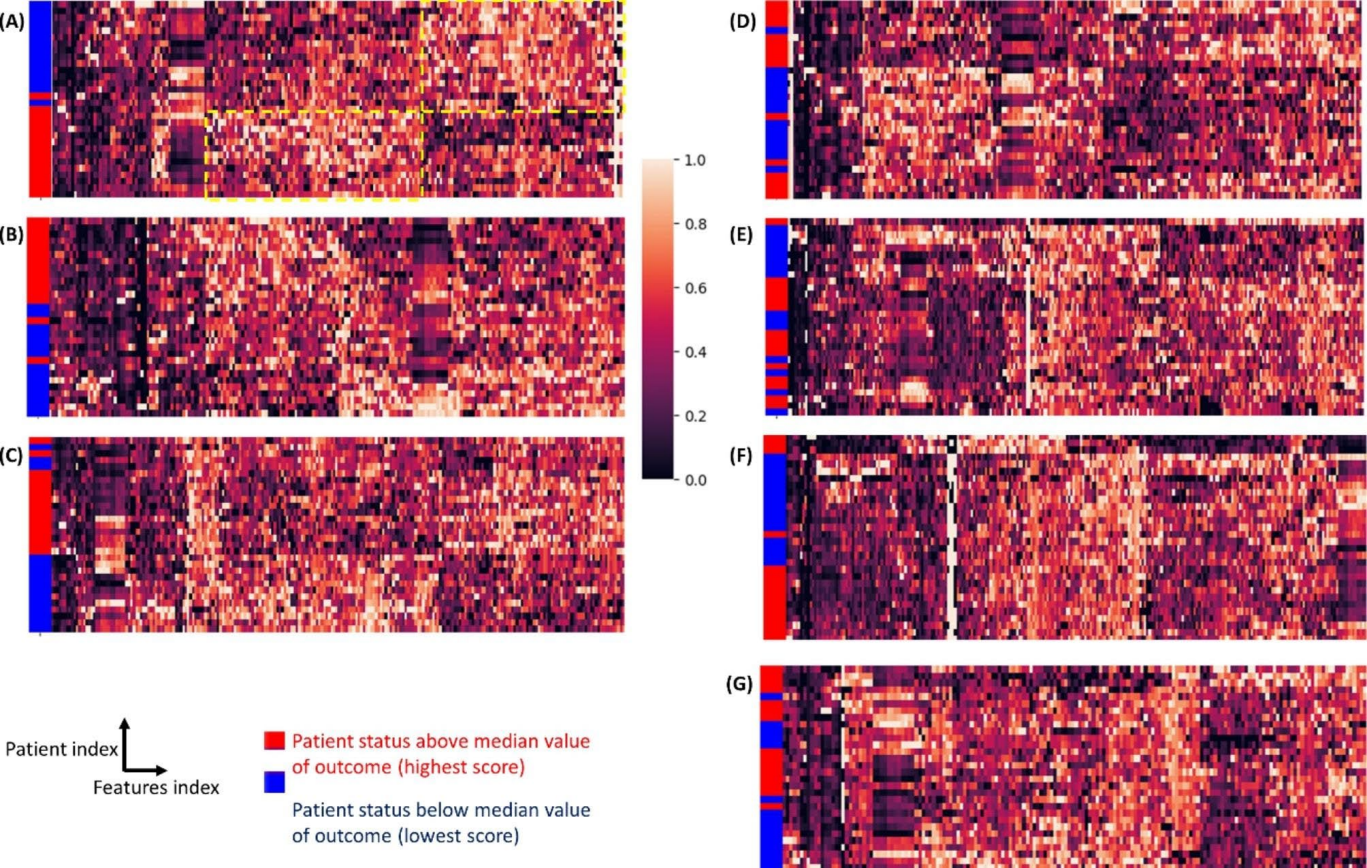
**Clustering patients according to functional status.** Agglomerative hierarchical clustering with standardized Euclidean distance metric and complete linkage method. Clustermap of 30 participants interfaced with PVs based on their similarity mapped into two groups below and above median value of each of the 7 outcomes: (A) 6MWT, (B) PROMIS fatigue score, (C) SWAY balance score, (D) BTHS-SA, (E) Muscle strength by HHD, (F) 5XSST, and (G) MLCL:CL. Yellow dotted line area in (A) represent the set of PVs with high expression level of similarity only for one group of functional status. 5XSST, 5 times sit-to-stand test; 6MWT, 6-minute walking test; BTHS-SA, Barth Syndrome Symptom Assessment; HHD, handheld dynamometry; MLCL:CL, monolysocardiolipin to cardiolipin ratio; PROMIS, Patient-Reported Outcomes Measurement Information System; PV, physiological variable

**Fig. 2:**
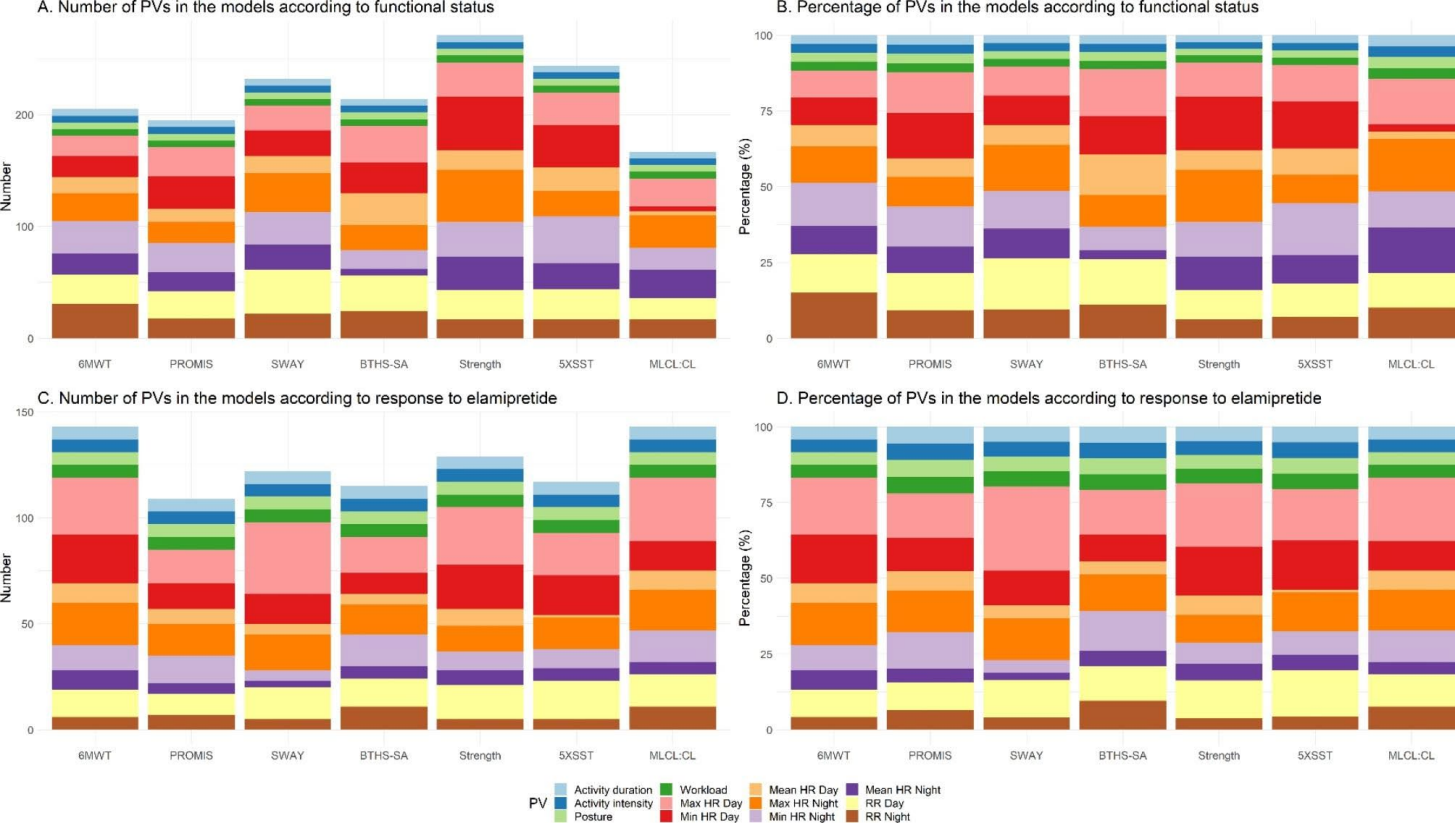
**Bar plots representing the numbers and percentages of PVs included each of the 10 agglomerative hierarchical clustering models.** 5XSST, 5 times sit-to-stand test; 6MWT, 6-minute walking test; AD, activity duration; AI, activity intensity; BTHS-SA, Barth Syndrome Symptom Assessment; HHD, handheld dynamometry; HR, heart rate; MLCL:CL, monolysocardiolipin to cardiolipin ratio; P, posture; PROMIS, Patient-Reported Outcomes Measurement Information System; PV, physiological variable; RR, respiratory rate; WL, workload

AHC models were implemented to map similar subjects into two clusters and interface with a set of PVs which were filtered with respect to an assessed outcome. These two clusters were then assessed with the true labels from the median split groups of the outcome variable to calculate AHC accuracy. The AHC models clustered the patients into groups above and below median value of outcome with accuracies ranging from 60 to 93% (Fig. 1; Table 1). The greatest accuracies were observed for 6MWT (93%), PROMIS fatigue score (87%), and SWAY balance score (80%). A mean of 218 PVs (range 167–271) were clustered with 30 observations (3 visits in 10 patients) to observe the distinguishable pattern observed in Fig. 1 (Table 1). The most commonly included PVs were those related to nighttime max HR (13.1%), nighttime min HR (12.7%), daytime RR (12.6%), daytime min HR (12.3%), and daytime max HR (12.0%) (Fig. 2A and B).

**Table 1:** **Performance of agglomerative hierarchical clustering models: according to functional status.** Performance metrics of three agglomerative hierarchical clustering models in clustering 30 observations (3 visits in 10 participants) with respect to their outcome value. 5XSST, 5 times sit-to-stand test; 6MWT, 6-minute walking test; BTHS-SA, Barth Syndrome Symptom Assessment; HHD, handheld dynamometry; MLCL:CL, monolysocardiolipin to cardiolipin ratio; PROMIS, Patient-Reported Outcomes Measurement Information System; PV, physiological variable

Outcome measure	True Negative	False Positive	False Negative	True Positive	Accuracy (%)	Number of PVs
6MWT	14	1	1	14	93	205
PROMIS Fatigue score	13	2	2	13	87	195
SWAY Balance score	12	3	3	12	80	232
BTHS-SA Total Fatigue	9	6	6	9	60	214
Muscle Strength by HHD	9	6	6	9	60	271
5XSST	11	4	4	11	73	244
MLCL:CL	10	5	5	10	67	167

### Physiological data accurately classify patients according to response to elamipretide

The second objective of the analysis was to apply hierarchical clustering to select features that can adequately distinguish non-responders from responders to elamipretide. The outcomes in this analysis were assessed by subtracting the baseline outcome (Base1 or Base2 depending on allocation) from elamipretide treatment outcome (End1 or End2 depending on allocation) to isolate the effect of elamipretide. Similar to the previous analysis, the outcomes were then split at the median to divide the 10 patients into responders and non-responders to elamipretide.

**Fig. 3:**
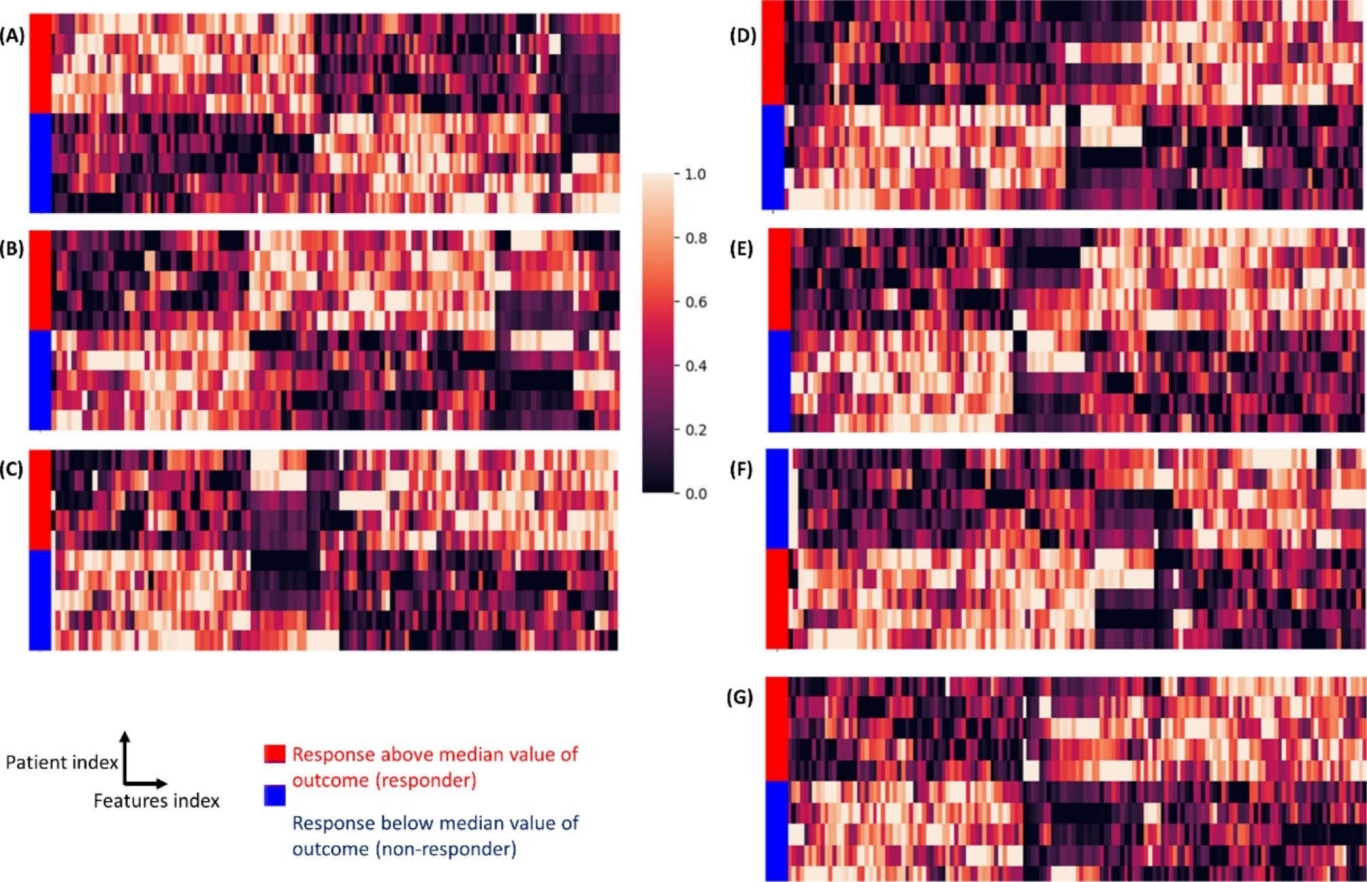
**Clustering patients according to response to elamipretide.** Agglomerative hierarchical clustering with standardized Euclidean distance metric and complete linkage method. Clustermap of 10 participants interfaced with PVs based on their similarity mapped into responders and non-responders groups to drug elamipretide. Clustermap of PVs with outcomes (A) 6MWT, (B) PROMIS fatigue score, (C) SWAY balance score, (D) BTHS-SA, (E) Muscle strength by HHD, (F) 5XSST, (G) MLCL:CL. 5XSST, 5 times sit-to-stand test; 6MWT, 6-minute walking test; BTHS-SA, Barth Syndrome Symptom Assessment; HHD, handheld dynamometry; MLCL:CL, monolysocardiolipin to cardiolipin ratio; PROMIS, Patient-Reported Outcomes Measurement Information System; PV, physiological variable

AHC models were then tailored to select PVs obtained from only elamipretide clinical visit data and map patients into two clusters. All AHC models have clustered the patients into groups with respect to their elamipretide treatment group response identity with a perfect accuracy score (all 100%) (Fig. 3; Table 2). It is clearly evident from all cases in Figs. 1 and 2 there exists a pattern with a specific set of PVs having a high expression level of similarity and correlating with only one group of patient status relative to the remaining patient group, which may adequately distinguish non-responders from responders to elamipretide. A mean of 125 PVs (range 109–143) were clustered with 10 patients to observe the clear distinguishable pattern between responder and non-responder groups observed in Fig. 3. The most commonly included PVs were those related to daytime max HR (19.5%), daytime min HR (12.9%), nighttime max HR (12.8%), daytime RR (11.4%), and nighttime min HR (8.9%) (Fig. 2 C-2D).

**Table 2:** **Performance of agglomerative hierarchical clustering models: according to response to elamipretide.** Performance metrics of three agglomerative hierarchical clustering models in clustering 10 participants with respect to their response to elamipretide for each of the outcomes. 5XSST, 5 times sit-to-stand test; 6MWT, 6-minute walking test; BTHS-SA, Barth Syndrome Symptom Assessment; HHD, handheld dynamometry; MLCL:CL, monolysocardiolipin to cardiolipin ratio; PROMIS, Patient-Reported Outcomes Measurement Information System; PV, physiological variable

Outcome measure	True Negative	False Positive	False Negative	True Positive	Accuracy (%)	Number of PVs
6MWT	5	0	0	5	100	143
PROMIS Fatigue score	5	0	0	5	100	109
SWAY Balance score	5	0	0	5	100	122
BTHS-SA Total Fatigue	5	0	0	5	100	115
Muscle Strength by HHD	5	0	0	5	100	129
5XSST	5	0	0	5	100	117
MLCL:CL	5	0	0	5	100	143

## Discussion

The present study showed that continuously acquired physiological measurements such as HR, RR, and activity-related metrics acquired using wearable devices can be used to predict (i) functional status and (ii) response to treatment with elamipretide in patients with BTHS. In the first part of the study, AHC models clustered patients according to their functional status with accuracies of 60–93% based on a mean of 218 PVs, with the greatest accuracies for 6MWT (93%), PROMIS fatigue score (87%), and SWAY balance score (80%). In the second part of the study, another set of AHC models clustered patients with respect to their response to treatment with elamipretide with perfect accuracy (all 100%) based on a mean of 125 PVs. Collectively, these findings suggest that the application of machine learning-based techniques to data from wearable devices may open new frontiers in patient phenotyping, monitoring and stratification.

While randomized controlled trials can be used to establish efficacy and safety of a therapy at the population level, at most they can generate data about the “average treatment effects” that would apply for an “average patient in the trial population” [20]. In reality, however, it is well agreed upon that a treatment’s effect varies across a population – a concept described as heterogeneity of treatment effects [21]. Individual patients may have many characteristics that potentially influence the benefit-harm ratio of a treatment and/or may affect the likelihood of an outcome. Only if these characteristics are being taken into account can optimal management of individual patients be achieved. Therefore, a major hurdle in the way of achieving true personalized medicine is to find ways of accurately classifying patients according to their expected treatment response.

With the widespread availability of wearable devices, a large amount of physiological data has become immediately accessible [22, 23]. These data represent a largely untapped resource in medicine, which could be leveraged for the realization of personalized medicine. Our present study provides a novel paradigm for how this could be achieved through a combination of time series data acquired from wearables and the clustering abilities of AHC models. Indeed, the time series data used in this study were gathered during normal daily activities, and when features derived from them were fed to AHC models, the latter were able to predict the (improvement in) performance of individual BTHS patients on 7 tests in a standardized test environment (i.e. 6MWT, PROMIS fatigue score, SWAY balance score). Our study thereby has incremental value beyond the elamipretide randomized controlled trial [11], demonstrating that those with the best response to elamipretide can be distinguished from those with the worst response to elamipretide based on continuous physiological measurements.

It is notable that certain PVs were more closely related to functional status and/or treatment response than others. Interestingly, for each of the AHC models, PVs based on max and mean HR values were more commonly included than those based on mean HR (Fig. 2). Max and min HR are probably more informative of an individual’s “fitness” (and thus, performance on the standardized tests), as they reflect the ability to respond to exercise and other physiological stressors. While the relative contributions of max and min HR differed between models, one striking observation could be made: max HR was the single most important contributor to the models for MLCL:CL. Accumulation of MLCL and loss of CL – and thus an increased MLCL:CL ratio – are directly related to lower tafazzin activity and more severe mitochondrial dysfunction. The close association with variations in maximal HR might therefore reflect restrictions in the ability to perform vigorous activities [15, 24]. While daytime and nighttime measurements contributed equally in the AHC models for functional status, daytime measurements were clearly predominant (about 60%) in the AHC models for treatment response. Since elamipretide is administered as a daily dose in the morning, this might have to do with plasma levels being lower overnight, leaving the greatest observable effect of therapy during the day. Another possible explanation is that elamipretide only has an effect on activities above a certain threshold, which is achieved only during the day. Finally, activity duration, activity intensity, posture, and workload were included in all models and were interpreted by the models in conjunction with the HR- and RR-based measurements because the latter are dependent on activity.

The major strength of this study is its innovative application of unsupervised machine learning (AHC models) to cluster patients according to their functional status or treatment response. The use of unsupervised machine learning to create a prediction model in a very rare disease is a novel approach as this is a substantial analytic challenge. Traditional machine learning approaches (including deep learning) usually require large datasets for algorithm training which tend to preclude their use in rare disease. Unsupervised machine learning, on the contrary, uses feature similarity between patients to cluster patients and uncover underlying patterns based on those clusters. This allows for prediction of outcomes for new patients based on their feature similarity to existing examples.

The models demonstrated excellent accuracy for a total of 7 different outcomes, covering a spectrum of important functional markers in patients with BTHS. Furthermore, an extensive set of features based was screened when constructing each of the AHC models, allowing for the most predictive ones to be selected. More specifically, the use of higher order features (derived using the “tsfresh” package) from time series data lends itself well for classification or clustering purposes, allowing to pack existing data variability within only a handful of features specific to different segments of the population. In addition, all time series data were obtained using the AVIVO™ mobile patient management system (Medtronic Inc.), which required no efforts from the patients except for wearing it for 7 consecutive days.

However, there are some limitations which need to be considered. First, because BTHS is an extremely rare disease, our study could only include 10 patients. Second, our study should be regarded as an exploratory proof-of-concept study. Since accuracy was calculated based on the same data that were used for training, our estimates might be optimistic, and external validation of our findings is warranted before clinical use. Third, while dichotomous endpoints based on median split were used (“highest value” versus “lowest value”), we did not test whether the exact values for functional status and treatment response could be predicted by the AHC models. Finally, we did not test whether data from the more commonly used smartwatches allowed for similarly accurate AHC models; this will require further investigation.

## Conclusions

In conclusion, the present study demonstrated that continuous physiological measurements from wearable devices can be used to predict functional status and response to treatment among patients with BTHS. This proof-of-concept thereby introduces a novel paradigm in machine learning that might allow for accurate classification of patient phenotypes and prediction of treatment responses in various domains of medicine.

## Data Availability

Original data generated and analyzed during this study belongs to the sponsor of the original trial and permission for access and use must be secured from them. The code used for analysis during the current study will be made available from the corresponding author on reasonable request.
